# Drug repositioning of Clopidogrel or Triamterene to inhibit influenza virus replication in vitro

**DOI:** 10.1371/journal.pone.0259129

**Published:** 2021-10-29

**Authors:** Nichole Orr-Burks, Jackelyn Murray, Kyle V. Todd, Abhijeet Bakre, Ralph A. Tripp

**Affiliations:** Department of Infectious Diseases, College of Veterinary Medicine, University of Georgia, Athens, GA, United States of America; University of South Dakota, UNITED STATES

## Abstract

Influenza viruses cause respiratory tract infections and substantial health concerns. Infection may result in mild to severe respiratory disease associated with morbidity and some mortality. Several anti-influenza drugs are available, but these agents target viral components and are susceptible to drug resistance. There is a need for new antiviral drug strategies that include repurposing of clinically approved drugs. Drugs that target cellular machinery necessary for influenza virus replication can provide a means for inhibiting influenza virus replication. We used RNA interference screening to identify key host cell genes required for influenza replication, and then FDA-approved drugs that could be repurposed for targeting host genes. We examined the effects of Clopidogrel and Triamterene to inhibit A/WSN/33 (EC_50_ 5.84 uM and 31.48 uM, respectively), A/CA/04/09 (EC_50_ 6.432 uM and 3.32 uM, respectively), and B/Yamagata/16/1988 (EC_50_ 0.28 uM and 0.11 uM, respectively) replication. Clopidogrel and Triamterene provide a druggable approach to influenza treatment across multiple strains and subtypes.

## Introduction

There are four types of influenza viruses (types A, B, C, and D) but only influenza A and B typically infect humans causing seasonal epidemics [1]. Influenza A virus (IAV) is divided into subtypes based on the hemagglutinin (HA) and neuraminidase (NA) genes. There are 18 different HA subtypes and 11 different NA subtypes. Influenza A subtypes are further divided into genetic clades. Influenza B viruses (IBV) are not divided into subtypes but are classified into two lineages, i.e. B/Yamagata and B/Victoria [1]. IAV and IBV are enveloped and contain eight negative-sense single-stranded RNA genome segments encoding 10 primary viral proteins (PB2, PB1, PA, HA, NP, NA, M1, M2, NS1, NS2) and various strain-dependent accessory proteins resulting from frameshift and alternative splicing events [2–7]. Seasonal epidemics arise from antigenic drift in the HA or NA surface proteins whereas pandemics are the result of viral genome reassortment events leading to vaccine failures [8, 9]. Young children, older individuals, and those immunocompromised are at greater risk of more serious influenza illness [10, 11]. Vaccines are the most effective available method of control against influenza disease; however, vaccine efficacy is variable within the population and may diminish in the event of strain mismatch [12, 13]. The CDC recommends four FDA-approved drugs (peramivir, zanamivir, oseltamivir, and baloxavir marboxil [14] for use against circulating influenza strains. Peramivir, zanamivir, and oseltamivir are neuraminidase (NA) inhibitors that impede influenza replication by inhibiting the budding of progeny virus from infected cells [14, 15]. The accumulation of point mutations within the NA gene during the 2007–2009 influenza season resulted in ~90% of circulating strains having oseltamivir resistance suggesting NAI overuse leads to drug resistance [16, 17]. Baloxavir marboxil inhibits the endonuclease activity of the polymerase acidic (PA) protein impeding viral RNA synthesis [18]. Unfortunately, baloxavir marboxil administration is nearly three times more expensive compared to oseltamivir [19], and as evidenced by a pediatric study in Japan, approximately 20% of the influenza viruses isolated from treated children developed mutations [20]. As of the 2004–2005 influenza season, amantadine and ramantadine, both M2 ion channel inhibitors, are no longer recommended due to increased resistance and limited efficacy [21, 22]. Taken together, this demonstrates a need for new disease intervention strategies and that target genes refractory to mutation such a cellular genes and pathways [23, 24]. As influenza virus is susceptible to drug-induced changes leading to resistance, targeting cell genes is recalcitrant to drug-induced changes or resistance. This approach also affords one the opportunity to develop combination therapies that may have a synergistic or additive effect when combined with current antivirals [25].

Traditional drug discovery involves structure-based screening or target-based screening approaches. Both methods can be cumbersome, lengthy, and costly. Approximately 0.1% of all compounds screened progress to an investigational new drug (IND) application, and less than half of these will move to phase III testing. The costs associated with research, development, and approval per IND are on average USD 4 billion and takes 10–15 years from preclinical testing to approval [26]. This bottleneck may be overcome by drug repurposing. In this study, we build upon previous RNA interference (RNAi) screening which identified host GPCR and ion channel targets required for influenza replication [27]. GPCRs are a superfamily of cell-surface receptor proteins that facilitate activation of intracellular signaling and downstream transcriptional events upon ligand binding [28]. Viruses usurp GPCRs to facilitate entry, replication, or egress [29–33]. Similarly, ion channels that facilitate Na^+^, K^+^, Cl^-^, or Ca^+2^ ion influx/efflux between the extracellular and intracellular compartments are used to modulate effector pathways and influenza replication [34, 35]. Based on the findings from GPCR and ion channel RNAi screens [27], we studied a panel of druggable host genes and focused on Clopidogrel and Triamterene as two repurposed drugs that inhibit influenza A (A/WSN/33 and A/CA/04/09), and influenza B (Yamagata/16/1988) replication. Host genes are potential pharmacological targets involved in virus replication.

## Results

### Clopidogrel and Triamterene

**Fig 1:**
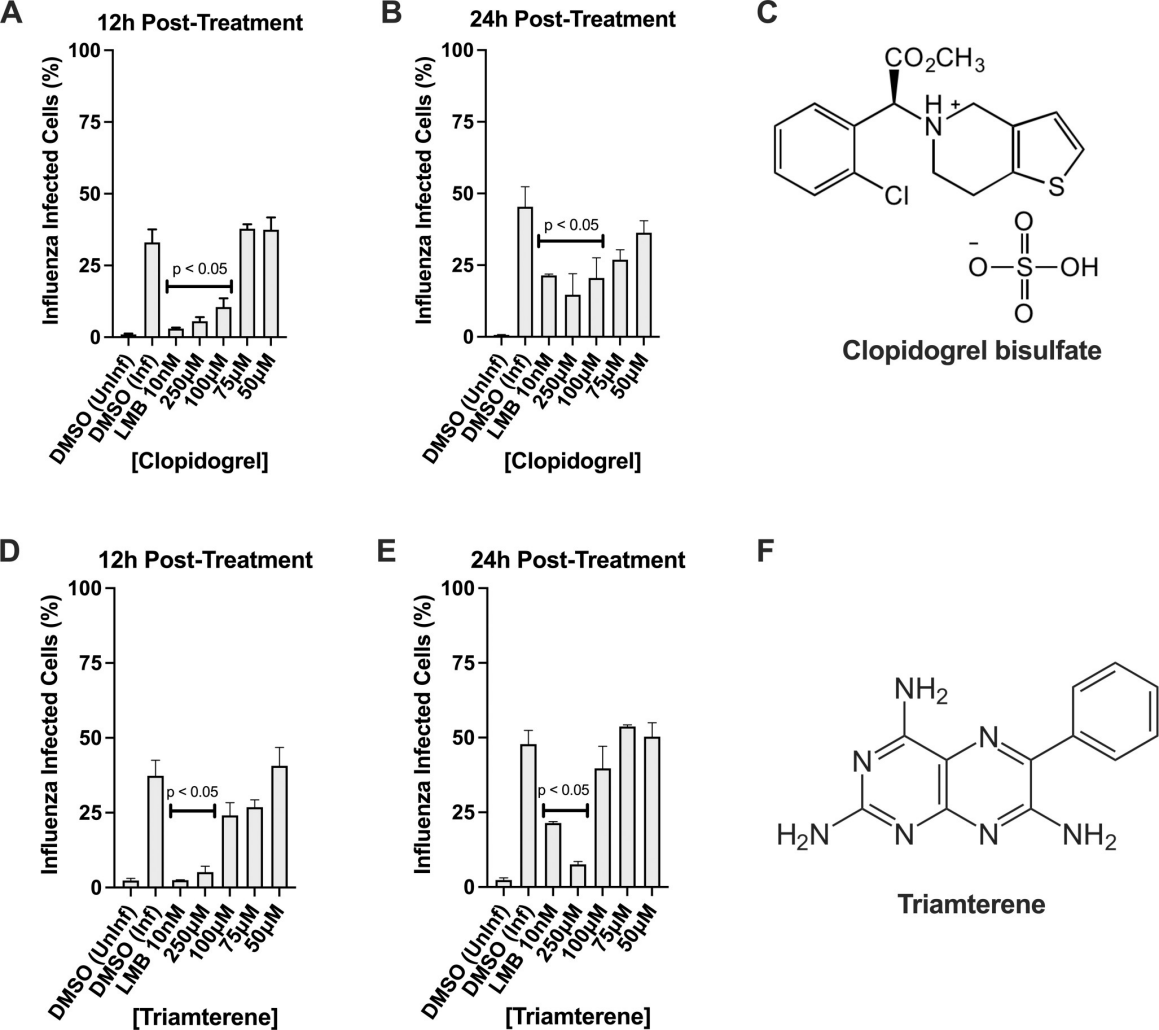
Clopidogrel and Triamterene have anti-influenza activity. A549 cells were infected for 1h before treatment with Clopidogrel for 12h (B) and 24h (C) or treated with Triamterene for 12h (E) and 24h (F) or LMB for 2h before infection after which the percent influenza-infected A549 cells was determined using an ArrayScan VTI HCS Reader and Cellomics Software. Presented are the structural formulas for compound SR-25990C, or Clopidogrel bisulfate (C) and compound SKF8542, or Triamterene (F). Shown is the mean ± standard errors for two independent experiments performed in triplicate. Statistically significant differences from DMSO-treated controls were determined by one-way analysis of variance with Dunnett’s multiple-comparison test (p<0.05) and are indicated on graphs where appropriate.

A dataset of 16 GPCR and 5 ion channel genes were previously identified and validated for replication of A/WSN/33, A/CA/04/09, and B/Yamagata/16/1988 influenza viruses [27]. We evaluated commercially available FDA-approved drugs that targeted either the GPCR or IC genes and short-listed this to 21 drugs or compounds. The drugs were purchased from SelleckChem or Tocris Bioscience and resuspended to 10 mM in DMSO. The drugs were screened against A/WSN/33 (MOI = 0.01) in a time of addition assay where A549 cells were infected for 1h before drug treatment. Leptomycin B (LMB) is used as the positive control as it has been previously shown to inhibit transport of vRNP from the nucleus reducing influenza virus replication and spread [25, 36]. The percentage of influenza-infected cells was determined at 12h and 24h post-treatment using influenza nucleoprotein as a marker for infection. Two drugs, Clopidogrel bisulfate (Fig 1C) and Triamterene (Fig 1F) reduced the percentage of influenza-infected A549 cells following treatment at 12 and 24 hours post-infection (hpi) (Fig 1). Clopidogrel significantly (p<0.05) reduced the percentage of influenza-infected cells at 12hpi in cells treated with 200 uM and 250 uM drug, and at 24hpi post-treatment with 250 uM drug (Fig 1A and 1B). Triamterene significantly (p<0.05) reduced the percentage of influenza-infected cells at 12hpi in cells treated with 250 uM drug, and markedly reduced virus replication when cells were treated with 75 or 100 uM drug (Fig 1D). Using 250 uM Triamterene, treatment significantly (p<0.05) reduced infection at 24hpi (Fig 1E). These results were not due to a loss of cell viability as determined by a CellTiter Blue assay (S1 Fig). Of note, the LMB control showed a significant reduction in NP+ cells at each time point (Fig 1).

#### Clopidogrel pretreatment inhibits A/WSN/33 replication in Calu-3 cells

**Fig 2:**
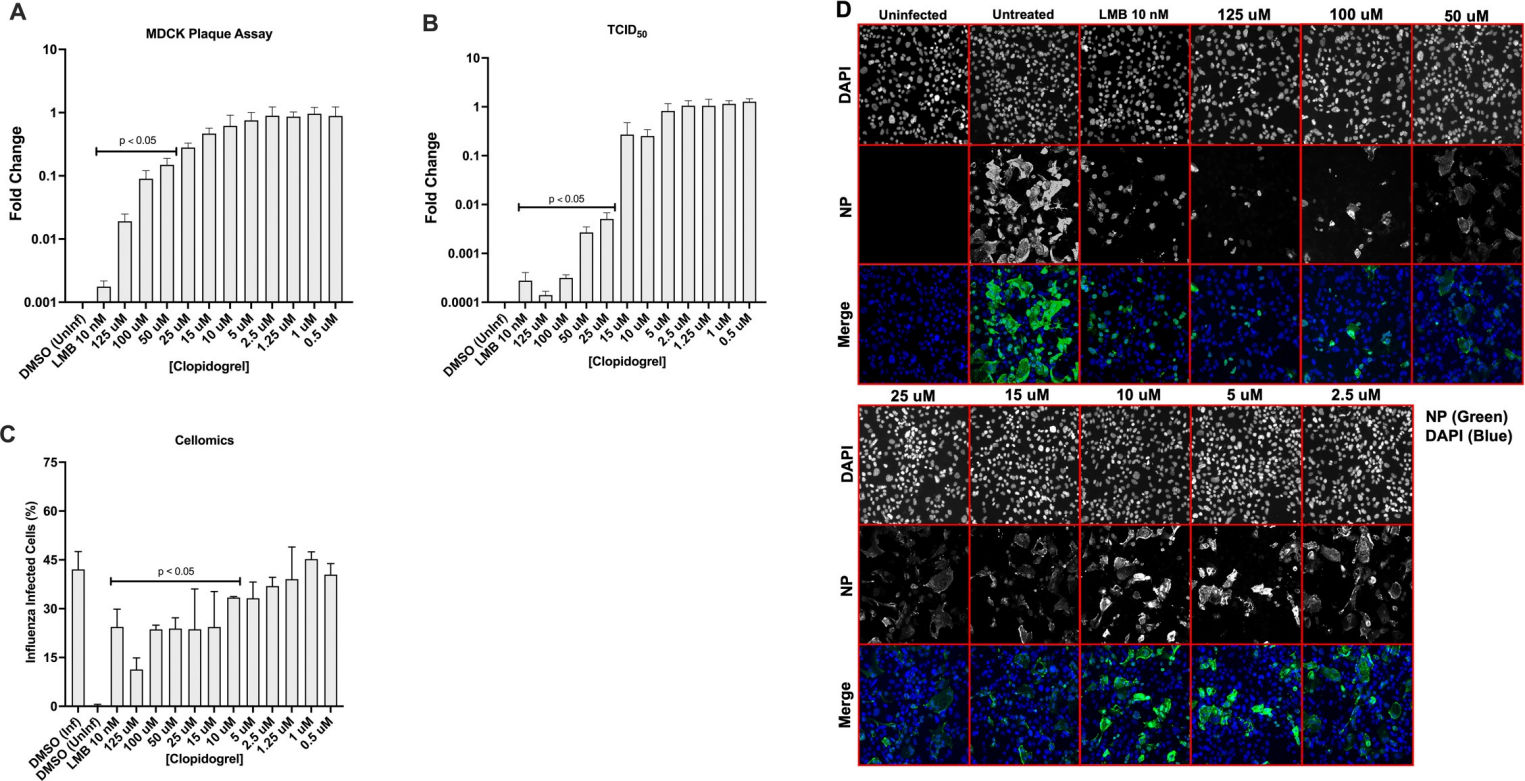
Pretreatment with Clopidogrel reduces A/WSN/33 replication. Calu-3 cells were treated with Clopidogrel for 24h or LMB for 2h before infection with A/WSN/33 (MOI = 0.01). At the time of infection, the media was removed and the virus was added with drug replenishment. The infection was incubated for 24h. Post-infection, supernatants were collected and evaluated by plaque assay (A) and TCID_50_ HA endpoint assay (B). Fixed Calu-3 cells were immunostained for NP and DAPI for Cellomics analysis (C) and imaging (D). Data show means ± standard errors of the means for two independent experiments performed in triplicate. Statistically significant differences from DMSO-treated controls were determined by one-way analysis of variance with Dunnett’s multiple-comparison test (p<0.05) and are indicated on graphs where appropriate. Influenza NP is green; nuclei are blue.

Calu-3 human bronchial epithelium cells are widely used for the evaluation of influenza and other respiratory viruses [37–39]. Calu-3 cells recapitulate the human lung compared to A549 cells and may provide more translatable results [40]. Clopidogrel was not cytotoxic to Calu-3 cells as determined by a CellTiter Blue assay (S2 Fig). We tested Clopidogrel for its ability to reduce A/WSN/33 replication in Calu-3 cells. Leptomycin B (LMB) is used as the positive control as it has been previously shown to inhibit transport of vRNP from the nucleus reducing influenza virus replication and spread [25, 36]. LMB had no detectable effect on cell viability (S2 Fig). Calu-3 cells were pretreated with Clopidogrel for 24h before infection with A/WSN/33 (MOI = 0.01). Calu-3 cells were pretreated for 2h with 10 nM LMB prior to A/WSN/33 (MOI = 0.01) infection. At the time of infection, the media was removed and replaced with A/WSN/33, infection media, and Clopidogrel and incubated for 24h. Following infection, the supernatants were collected and evaluated by plaque assay and TCID_50_ HA assay. Calu-3 cells were fixed with acetone/methanol, immunostained for influenza nucleoprotein (NP), and DAPI stained for Cellomics analysis (Fig 2). Influenza virus titers were determined by titration of sample supernatants on MDCK cell monolayers as described [27, 41, 42]. Compared to DMSO-treated controls, Clopidogrel pretreatment significantly (p<0.05) reduced influenza titers using 50–125 uM concentrations resulting in an EC_50_ of 5.8 uM (Fig 2A). The fold-change in TCID_50_ HA titer was also significantly (p<0.05) reduced using 25–125 uM concentrations compared to the control (Fig 2B). The LMB control had a significant (p<0.05) decrease in fold-change compared to the DMSO control for influenza titer (PFU) and TCID_50_ HA titer (Fig 2A and 2B). The percent influenza NP positive cells was determined using an Arrayscan comparing nuclei (DAPI) to NP, i.e. AlexaFluor488 stained cells. Cells were determined to be NP positive if their average intensity within the cytoplasm was higher than the control threshold. The findings showed that Clopidogrel treatment significantly (p<0.05) reduced influenza-infected cells using 15–125 uM concentrations (Fig 2C and 2D). Of note, at higher concentrations, Clopidogrel staining localized with DAPI staining suggesting nuclear retention of vRNPs (Fig 2D). These results show that Clopidogrel pretreatment effectively inhibits A/WSN/33 replication in Calu-3 cells.

#### Triamterene pretreatment inhibits A/WSN/33 replication in Calu-3 cells

**Fig 3:**
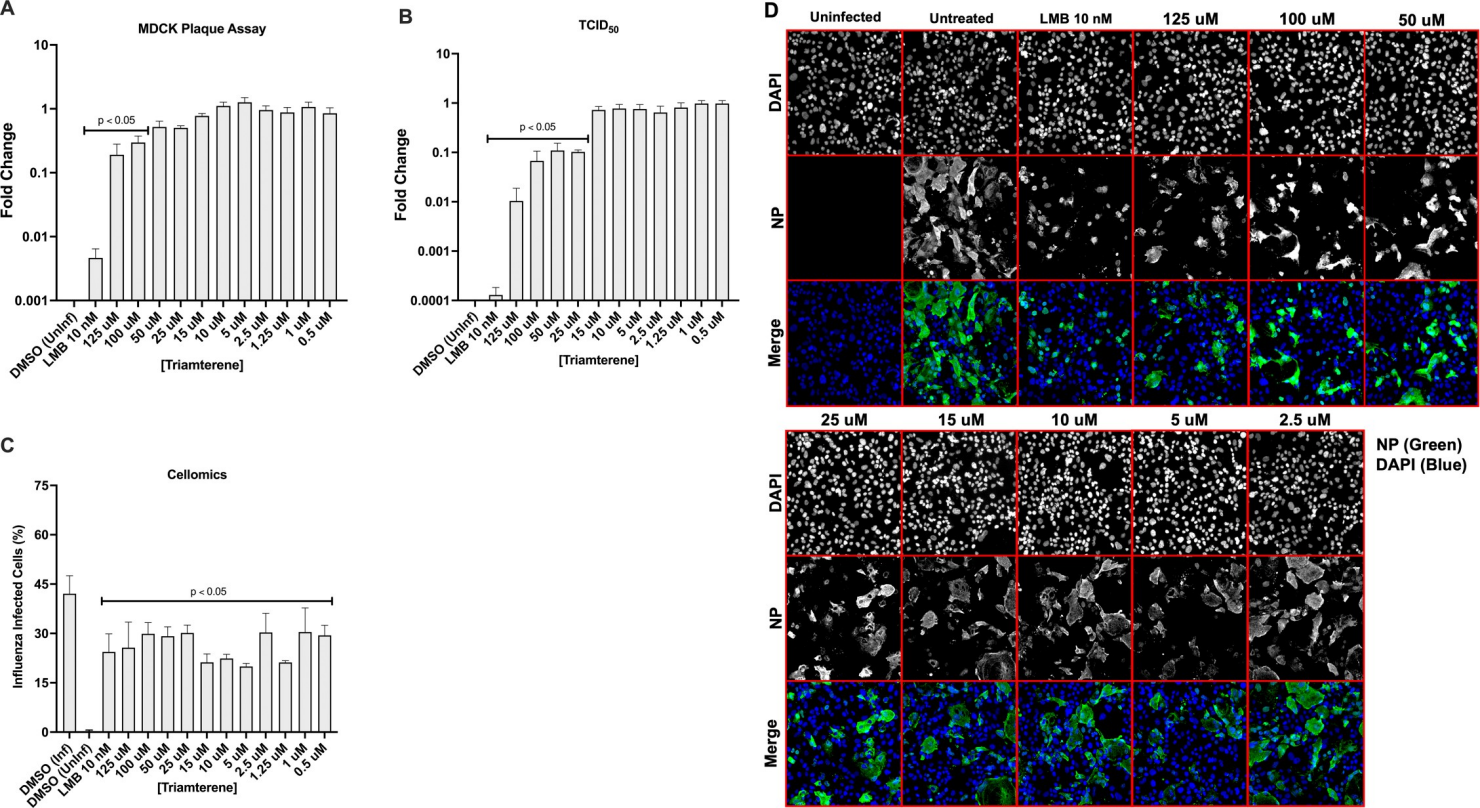
Pretreatment with Triamterene reduces A/WSN/33 replication. Calu-3 cells were pretreated with Triamterene for 24h, or LMB for 2h before infection with A/WSN/33 (MOI = 0.01). At the time of infection, the media was removed and the virus was added with drug replenishment. The infection was incubated for 24h. Post-infection, supernatants were collected and evaluated by plaque assay (A) and TCID_50_ assay with HA endpoint (B). Fixed Calu-3 cells were immunostained for NP and with DAPI for Cellomics analysis (C) and imaging (D). The results were determined as means ± standard errors of the means for two independent experiments performed in triplicate. Statistically significant differences from DMSO-treated controls were determined by one-way analysis of variance with Dunnett’s multiple-comparison test (p<0.05) and are indicated on graphs where appropriate. Influenza NP is green; nuclei are blue.

Using Calu-3 cells, we evaluated Triamterene for antiviral activity. Triamterene was not cytotoxic for Calu-3 cells using a CellTiter Blue assay (S3 Fig). Calu-3 cells were pretreated with Triamterene for 24h before A/WSN/33 (MOI = 0.01) infection. As a positive control, Calu-3 cells were pretreated for 2h with 10 nM LMB before A/WSN/33 (MOI = 0.01) infection. At the time of infection, the media was removed and replaced with A/WSN/33 and Triamterene. The virus infection was incubated for 24h and supernatants were collected and evaluated by plaque assay and TCID_50_ HA assay. Calu-3 cells were fixed with acetone:methanol, immunostained for influenza NP, and stained with DAPI for Cellomics assays (Fig 3). Triamterene pretreatment significantly (p<0.05) reduced influenza plaque titers using 100–125 uM concentrations resulting in a EC_50_ of 31.5 uM. Concentrations ranging from 25–125 uM significantly (p<0.05) reduced TCID_50_ HA titer (Fig 3A, 3C, and 3D). The influenza titers for the LMB control were also significantly (p<0.05) reduced (Fig 3A and 3B). These results show that Triamterene pretreatment effectively inhibits A/WSN/33 replication in Calu-3 cells.

#### The antiviral activity of Clopidogrel is not influenza virus strain or type-specific

**Fig 4:**
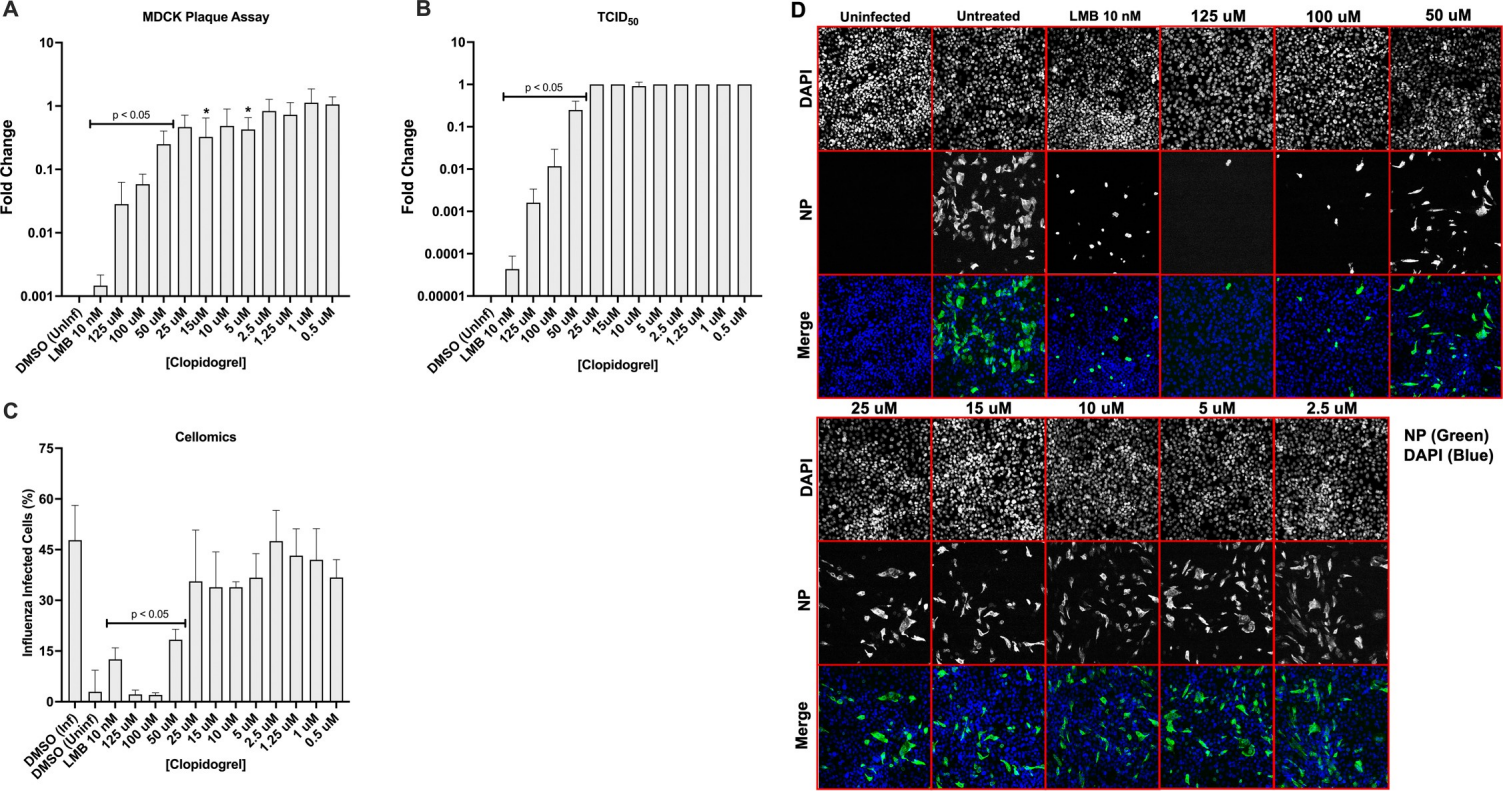
Pretreatment with Clopidogrel reduces A/CA/04/09 and B/Yamagata/16/1988 replication. Calu-3 cells were treated with Clopidogrel for 24h, or with LMB for 2h before infection with either A/CA/04/09 (MOI = 0.1) (A-D) or B/Yamagata/16/1988 (MOI = 0.1) (E-H). At the time of infection, the media was removed and the virus was added with drug replenishment. The infection was incubated for 24h. Post-infection, supernatants were collected and evaluated by plaque assay (A, E) and TCID_50_ assay with HA endpoint (B, F). Fixed Calu-3 cells were immunostained for NP (C, D) or B/Yamagata/16/1988 antigen (G, H) and with DAPI for Cellomics analysis and imaging. Data show means ± standard errors of the means for two independent experiments performed in triplicate. Statistically significant differences from DMSO-treated controls were determined by one-way analysis of variance with Dunnett’s multiple-comparison test (p<0.05) and are indicated on graphs where appropriate. Influenza NP is green; nuclei are blue.

Clopidogrel is a small molecule inhibitor of P2RY12, a GPCR gene [43–45]. We have previously shown that RNAi silencing of P2RY12 reduces influenza replication [27]. Associated with P2RY12 is Gα_i_ signaling and activation of the Raf/MEK/Erk pathway [46]. Inhibition of the Raf/MEK/Erk cascade during influenza infection leads to reduced influenza virus production and retention of vRNP within the nucleus [47, 48]. We sought to determine if the antiviral activities of Clopidogrel were influenza strain or type-specific. Calu-3 cells were pretreated with Clopidogrel for 24h or with 10 nM LMB for 2h before infection with A/CA/04/2009 (MOI = 0.1). At the time of infection, the media was removed and A/CA/04/2009 was added and Clopidogrel replenishment. The infection was incubated for 24h, and the cell supernatants were collected at 24hpi and evaluated by plaque assay and TCID_50_ HA assay. Calu-3 cells were fixed with acetone:methanol, immunostained for influenza NP, and stained with DAPI for Cellomics assays (Fig 4A–4D). Clopidogrel pretreatment significantly (p<0.05) reduced A/CA/04/09 titers using 50–125 uM concentrations having an EC_50_ of 6.4 uM (Fig 4A). TCID_50_ HA titers were significantly (p<0.05) reduced using 50–125 uM concentrations (Fig 4B). As expected, the LMB control significantly (p<0.05) reduced for both influenza titer and TCID_50_ HA titer (Fig 4A and 4B). The percentage of influenza-infected cells was significantly (p<0.05) reduced using 50–125 uM concentrations, and markedly reduced using 2.5–25uM treatment (Fig 4C and 4D). As observed earlier, NP staining localized with DAPI at higher Clopidogrel treatment concentrations suggesting nuclear retention of vRNPs (Fig 4D).

To determine if the antiviral activity of Clopidogrel was influenza type-specific it was evaluated against B/Yamagata/16/1988 for antiviral efficacy. Calu-3 cells were pretreated with Clopidogrel for 24h, or LMB 2h, before infection with B/Yamagata/16/1988 (MOI = 0.1), incubated for 24h and evaluated by plaque assay and TCID_50_ assay. Clopidogrel pretreatment reduced B/Yamagata/16/1988 titers from 1.2–125 uM concentrations resulting in an EC_50_ of 0.3 uM (Fig 4E). Pretreatment significantly (p<0.05) reduced the HA titer and at 50–125 uM concentrations there was a greater reduction of TCID_50_ titer compared to LMB (Fig 4F). The LMB control reduced influenza titer and TCID_50_ HA titer (Fig 4E and 4F). The percentage of influenza-infected cells was consistently reduced compared to untreated control with all concentrations and significantly (p<0.05) reduced with nearly all concentrations of drug (Fig 4G and 4H). These data show broad antiviral effects of Clopidogrel treatment against host genes used by influenza to aid viral replication of influenza A and B strains.

#### The antiviral activity of Triamterene is not influenza virus strain or type specific

**Fig 5:**
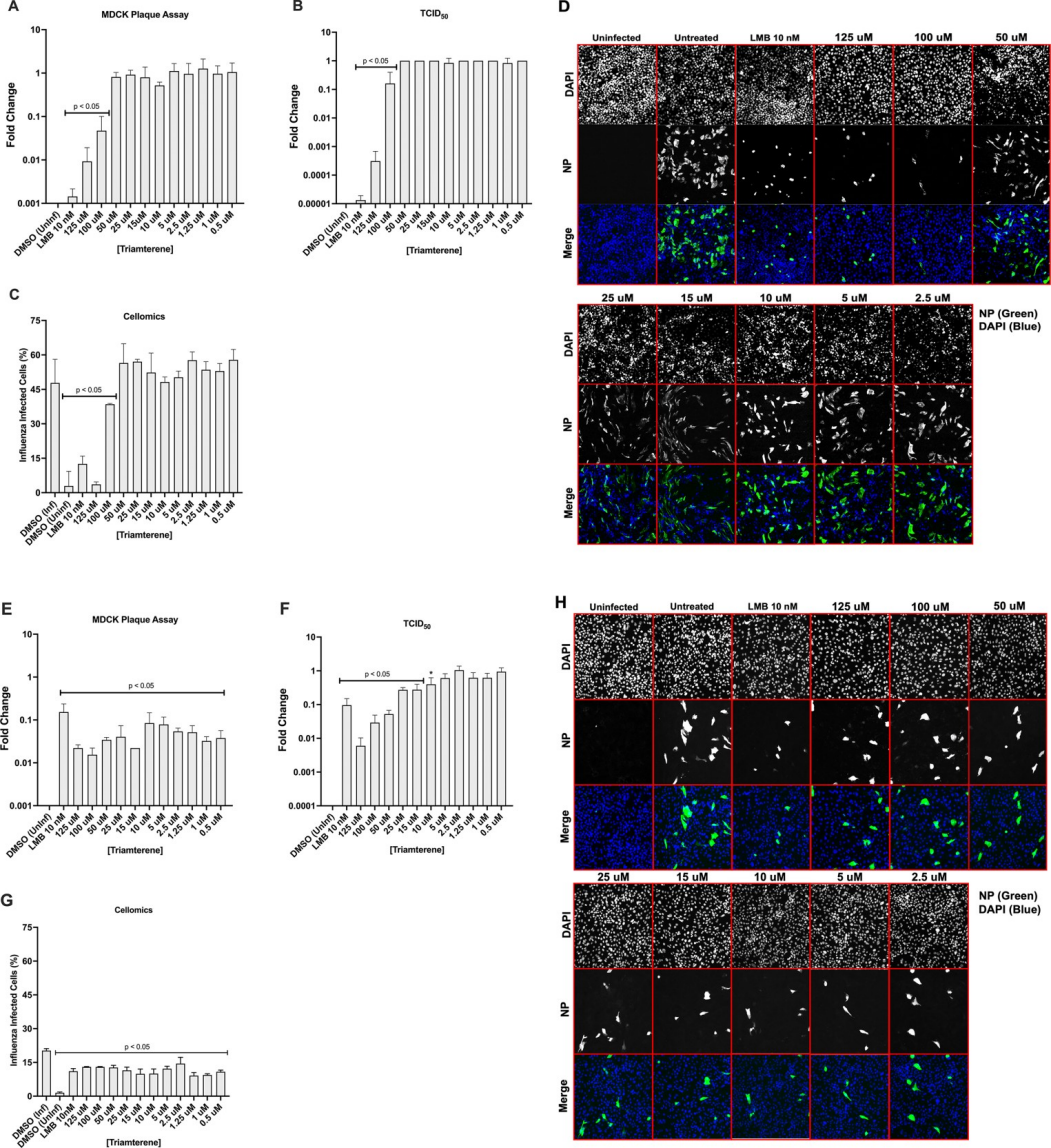
Pretreatment with Triamterene reduces A/CA/04/09 and B/Yamagata/16/1988 replication. Calu-3 cells were pretreated with Triamterene for 24h, or LMB for 2h before infection with either A/CA/04/09 (MOI = 0.1) (A-D) or B/Yamagata/16/1988 (MOI = 0.1) (E-H). At the time of infection, the media was removed and the virus was added with drug replenishment. The infection was incubated for 24h. Post-infection, supernatants were collected and evaluated by plaque assay (A, E) and TCID_50_ assay with HA endpoint (B, F). Fixed Calu-3 cells were immunostained for NP (C, D) or B/Yamagata/16/1988 antigen (G, H) and with DAPI for Cellomics analysis and imaging. Data show means ± standard errors of the means for two independent experiments performed in triplicate. Statistically significant differences from DMSO-treated controls were determined by one-way analysis of variance with Dunnett’s multiple-comparison test (p<0.05) and are indicated on graphs where appropriate. Influenza NP is green; nuclei are blue.

Triamterene affects the expression of SCNN1 (or δENaC), a sodium channel host gene located on lung cells that are involved in sodium ion transport and reabsorption [49, 50]. We have previously shown that RNAi silencing of the SCNN1D reduces influenza replication [27]. We examined Triamterene for its ability to inhibit influenza replication across strains and types (Fig 5). Calu-3 cells were pretreated with Triamterene for 24h, or LMB 2h, before infection with A/CA/04/2009 (MOI = 0.1), incubated for 24h, and assayed evaluated by plaque assay and TCID_50_ assay. Triamterene pretreatment using 100–125 concentrations significantly (p<0.05) reduced A/CA/04/2009 titer resulting in an EC_50_ of 3.3 uM. TCID_50_ was also significantly reduced at these using 100–125 uM concentrations (Fig 5A and 5B). The LMB control significantly (p<0.05) as expected reduced influenza titer (PFU/ml) and TCID_50_ HA titer (Fig 5A and 5B). The percentage of influenza-infected cells was significantly (p<0.05) reduced using 50–125 uM concentrations (Fig 5C and 5D).

Triamterene efficacy was evaluated against B/Yamagata/16/1988 infection. Calu-3 cells were pretreated with Triamterene 24h, or LMB 2h before infection with B/Yamagata/16/1988 (MOI = 0.1), incubated for 24h, and assayed evaluated by plaque assay and TCID_50_ assay. Triamterene pretreatment significantly (p<0.05) reduced B/Yamagata/16/1988 titers at all concentrations tested resulting in an EC50 of 0.1 uM (Fig 5E). Pretreatment of Calu-3 cells with Triamterene for 24h led to a significant (p<0.05) dose-dependent reduction in TCID_50_ HA titer using 10–125 uM concentrations compared to the LMB control. (Fig 5E and 5F). The percentage of influenza-infected cells was significantly (p<0.05) reduced at all concentrations (Fig 5G and 5H). These data show that pretreatment with Triamterene for 24h can reduce A/CA/04/09 and B/Yamagata/16/1988 replication affirming the pan anti-influenza effects of Triamterene.

## Materials and methods

### Cells and viruses

Type II human lung epithelial (A549) cells (ATCC CCL-185) and Madin-Darby Canine Kidney (MDCK) cells (ATCC CCL-34) were maintained in Dulbecco’s modified Eagle’s Medium (DMEM; HyClone, Logan, UT) supplemented with 5% heat-inactivated fetal bovine serum (HI-FBS; Atlas Biologics Inc., Fort Collins, CO). Calu-3 lung epithelium cells (ATCC HTB-55) were propagated in DMEM containing 10% HI-FBS, 1% nonessential amino acid solution, 1% L-glutamine solution, and 1% HEPES solution (all from GIBCO). Cells were propagated as described [27].

A/WSN/33 (H1N1; ATCC VR-825) is a lab-adapted, trypsin-independent influenza A strain. A/CA/04/2009 (H1N1, BEI Resources) is a circulating influenza A strain. Both viruses were propagated in MDCK cells [51, 52]. B/Yamagata/16/1988 (BEI Resources), an influenza B strain was propagated in 9-day old embryonated chicken eggs as previously described [51]. Viral titer (PFU/ml) was determined by plaque assay using MDCK cells and the titer calculated using the Reed and Muench method [41, 53, 54].

### Identification of drugs

GPCR and ion channel genes targeted by the repositioned drugs were identified using Ingenuity Pathway Analysis (IPA, Qiagen, CA), Drugbank 3.0, the Drug Gene Interaction Database (DGIdg), and PubChem literature searches. Briefly, IPA, Drugbank, and DGIdg were used to identify potential reverse agonists/inhibitors and antagonist drugs targeting validated pro-influenza GPCR and ion channel genes based on data extracted from public databases [55–57]. Stringency filters were limited to drugs with direct reverse agonist/inhibitor or antagonistic action, human specificity, experimental evaluation, and current FDA approval. Commercially available drugs were purchased from SelleckChem (Houston, TX) or Tocris Bioscience (Bristol, UK). Drug stocks were prepared in DMSO to a stock concentration of 10 mM, aliquoted into working volumes, and stored at -20°C until needed. Drug stocks were discarded following freeze-thaw.

### CellTiter Blue viability assay

The selected drugs were evaluated for cytotoxicity using A549 cells and Calu-3 cells using a CellTiter blue viability assay (Promega, WI). Drugs that did not cause cytotoxicity when compared to the DMSO control (<20%) were further evaluated. Briefly, 1.5 x 10^4^ A549 cells or Calu-3 cells were seeded into 96-well flat-bottom plates (Costar) and incubated at 37°C/5% CO_2_. Subsequently, the cells were gently washed 1x with PBS (GIBCO), and minimum essential media (MEM; HyClone, Logan, UT) supplemented with 0.3% (v/v) bovine serum albumin (BSA; Gibco, Waltham, Massachusetts) which was added to the plates at 37°C/5% CO_2_. Drug stocks were prepared in filter-sterilized DMSO (Sigma) to a stock concentration of 10 mM. Drugs were dispensed into 96-well plates using a D300 BioPrinter digital drug dispenser (HP, Palo Alto, CA) in MEM supplemented with 0.3% BSA to final concentrations of 500, 200, 150 and 100 for A549 cells and 250, 200, 150, 100, 50, 30, 20, 10, 5, 2.5, 2 and 1 uM for Calu3 cells. Dilutions were transferred to A549 or Calu-3 plates for final drug concentrations of 250, 100, 75, and 50 uM and 125, 100, 75, 50, 25, 15, 10, 5, 2.5, 1.25, 1, and 0.5 uM, respectively. All wells were normalized to 1% DMSO for A549 cell evaluation, and 0.833% DMSO for Calu-3 cell evaluation. Cells were incubated for the relevant time points at 37°C/5% CO_2_. Following incubation, CellTiter blue was added to each well and incubated at 37°C/5% CO_2_ for 2 h. The absorbance of the plates was determined using a spectrophotometer plate reader (Tecan Trading; AG, Switzerland) at 570 nm with reference at 600 nm. Percent viability was determined by comparing the DMSO control to drug-treated cells.

### Drug screening

#### Time of addition

96-well flat-bottom plates (Costar) were seeded with 1.5 x 10^4^ A549 cells/well and incubated overnight at 37°C/5%CO_2_. Clopidogrel and Triamterene were prepared in DMSO to a stock concentration of 10 mM. Drugs plates were prepared using the D300 BioPrinter digital drug dispenser (HP, Palo Alto, CA) and dispensed into 96-well plates containing virus infection media containing MEM supplemented with 0.3% BSA and TPCK-treated trypsin (1 ug/ml) (Worthington, Lakewood, New Jersey) to the final concentrations 500, 200, 150, or 100 uM. A/WSN/33 virus (MOI = 0.01) was prepared in infection media. A549 cell plates were washed 2x with PBS. Equal volumes of infection and drug dilutions were transferred to the A549 cells (excluding control wells) to infect cells (MOI = 0.01) and achieve final concentrations of 250, 100, 75, or 50 uM of the drug. The wells were normalized to 1% DMSO. The plates were incubated for 12h or 24h at 37°C/5% CO_2_. Following incubation, the supernatants were removed, the plates were washed 2x with PBS, and the cells fixed with acetone:methanol (20:80; Sigma). Leptomycin B (LMB) (Sigma, St. Louis, MO) was used as the positive control for inhibition of influenza replication and was administer on cells 2h prior to infection [58, 59].

#### Prophylactic treatment with Clopidogrel and Triamterene

Calu-3 cells were plated in 96-well plates at 1.5 x 10^4^ cells per well and incubated overnight at 37°C/5% CO_2_. Clopidogrel and Triamterene were prepared in DMSO to a stock concentration of 10mM. Drugs plates were prepared by the D300 BioPrinter digital drug dispenser and dispensed into 96-well plates containing 200 ul DMEM (HyClone) plus with 4% BSA (GIBCO) to the final concentrations of 125, 100, 50, 25, 15, 10, 5, 2.5, 1.25, 1 and 0.5 uM. All wells were normalized to 0.833% DMSO. Plates were incubated with the drug for 24h at 37°C/5% CO_2_. Following incubation, a new 96-well plate was prepared where the drugs in infection media plus TPCK-treated trypsin were added at the final concentrations of 250, 200, 100, 50, 30, 20, 10, 5, 2.5, 2, and 1 uM. Either A/WSN/33 (MOI = 0.01), A/CA/04/09 (MOI = 0.1), or B/Yamagata/16/1988 (MOI = 0.1) were diluted in infection media and added to the wells of the new drug plate excluding the control wells bringing the final concentrations of drugs to 125, 100, 50, 25, 15, 10, 5, 2.5, 1.25, 1, and 0.5 uM, and incubated for 24h at 37°C/5% CO_2_. Following incubation, supernatants were collected and stored at -80°C until tested by plaque assay and TCID_50_ assay. A/WSN/33 and A/CA/04/09 infected cells were fixed in acetone:methanol (20:80) and B/Yamagata/16/1988 infected cells were fixed with 4% formalin in PBS and permeabilized with 0.5% Triton X-100 prior to staining for Cellomics analysis. Leptomycin B (LMB) (Sigma, St. Louis, MO) was used as the positive control for inhibition of influenza replication and was administered to cells 2h prior to infection [58, 59].

#### Cellomics

A549 cells and Calu3 cells infected with either A/WSN/33 or A/CA/04/09 were fixed in acetone:methanol (20:80) for 10 min and stained with murine anti-NP IgG (National Cell Culture Center, Minneapolis, MN) and DAPI (Invitrogen, Carlsbad, CA). Calu3 cells infected with B/Yamagata/16/1988 were fixed in 4% formalin in PBS for 20 min then permeabilized with 0.5% Triton X-100 for 10 min prior to staining with ferret sera raised against B/Yamagata/16/1988 and DAPI. The percentage of NP+ cells was quantified using an Arrayscan VTI HCS Reader and Cellomics software (Thermo Fisher, Waltham, MA). Briefly, for NP staining the fixed cells were blocked with Blotto (4% BSA fraction + 4% dry milk in KPL buffer) for 1h at room temperature. The blocking solution was decanted, replaced with murine anti-NP IgG in Blotto, and incubated at room temperature for 1h. Following the incubation, the antibody was removed, and plates washed 3x with KPL buffer, and then a secondary antibody goat anti-mouse IgG AlexaFluor488 (Invitrogen, Carlsbad, CA) in Blotto was added to the wells and incubated at room temperature for 1h. Briefly, for B/Yamagata staining fixed cells were blocked with Blotto (3 fixed cells were blocked with Blotto (3% BSA (Cohn fraction) in KPL buffer) for 1h at 37°C. The blocking solution was decanted, replaced with ferret B/Yamagata/16/1988 anti-sera in Blotto, and incubated at 37°C for 1h. Following the incubation, the antibody was removed, and plates washed 3x with KPL buffer, and then a secondary antibody goat anti-ferret IgG FITC (Abcam, Cambridge, UK) in Blotto was added to the wells and incubated at 37°C for 1h. In all cases following incubation, the antibody was removed, and plates were washed 3x with KPL buffer. The plates were stained with DAPI in PBS for 20 min and subsequently washed 2x with PBS. Following washing, PBS was added to each well and the percentage of NP+ cells was quantified using the Cellomics software, i.e. green (AlexaFluor488) and blue (DAPI). Background fluorescence was determined, and the baselines set using antibody-treated uninfected control wells. Images were captured at 10x magnification providing 49 fields per well for analysis. NP+ cells were determined using DAPI staining (nucleus) to identify all cells within the field and then used AlexaFluor488 or FITC staining (cytoplasm) to evaluate the cell area. The average intensity of AlexaFluor488 or FITC staining was quantified and compared to negative control. Cells were positive for infection if their average intensity for AlexaFluor488 or FITC within the cytoplasmic mask was higher than 3 standard deviations from the mean of the control.

#### Influenza plaque assay

MDCK cells were used in plaque assays to determine viral titers [27, 42, 53, 60]. Briefly, supernatants were serially diluted 10-fold in MEM with 1 ug/ml TPCK-trypsin and inoculated onto 90% confluent MDCK cell monolayers in 12-well tissue culture plates (Costar). The virus was adsorbed for 1h at 37°C/5% CO_2_ before adding 3 ml of an overlay. Overlay media contained 1-part liquid medium containing: 10x MEM supplemented with 200 mm L-glutamine, HEPES solution, 7.5% NaCHO_3_, Pen/Strep/Amp B solution (all from Gibco, Waltham, MA), and 1% agarose (Sigma) in water. Samples from A/WSN/33 or A/CA/0409 wells were incubated at 37°C/5% CO_2_ for 3 days. B/Yamagata/16/1988 infected cells were incubated at 37°C/5% CO_2_ for 5 days to allow for improved plaque formation. Following incubation, the plates were washed 2x with PBS, and the cell monolayers were fixed with methanol: acetone (80:20) for 20 min at room temperature. Following fixation, the plates were stained with 0.2% crystal violet (Fisher Scientific, Waltham, MA) as described to determine the virus titers [27, 42, 53, 60].

#### TCID_50_ HA assay

Endpoint titers were determined by TCID_50_ [27, 41, 61]. Briefly, supernatants that were collected from influenza virus-infected cells were serially diluted 10-fold in triplicate on MDCK cells in 96-well plates (Costar). Influenza virus-infected MDCK plates were incubated for 5 days using cell culture conditions as described [41, 53]. Following incubation, an HA test was performed using the supernatants from influenza virus-infected MDCKs and 0.5% turkey red blood cells in round-bottom plates (Costar) [61]. The TCID_50_ titers were calculated using the Reed and Muench method [41].

## Discussion

Influenza antiviral drugs may reduce virus replication but this strategy often leads to virus resistance [23, 24]. Thus, it is important to consider new strategies to combat influenza such as targeting host genes needed for replication [25, 36, 62, 63]. Host genes used for virus replication are recalcitrant to resistance and may provide the potential for pan-antiviral therapies as often viruses usurp the same or similar host pathways for replication [27]. In addition, drug discovery is time-consuming, costly, and fraught with failure. In contrast, drug repurposing can mitigate bottlenecks associated with drug discovery and generally reduces the time and cost for implementation [64]. We previously utilized RNAi host gene screens to identify GPCR and ion channel genes required for influenza A and B virus replication [27, 55–57], and to identify drugs for repurposing. Our host gene pathway analysis led us to investigate two FDA-approved drugs, i.e. Clopidogrel and Triamterene, which when tested prophylactically reduced influenza A and B virus replication in human respiratory epithelial cell lines, i.e. A549 and Calu-3 cell lines.

GPCRs represent the largest drug target family, and ion channels are the second largest of approved drugs [65, 66]. The genes facilitate the activation and modulation of host pathways often hijacked by viruses to facilitate entry, replication, and egress [32, 33]. For example, replication of the Marburg virus and Ebola virus replication is linked to GPCR usage [29–31, 67], while modulation of specific ion channels reduces Bunyamvera virus, herpes simplex virus-1, and influenza A viruses [34, 68–70]. Furthermore, ion channels affect the efficient viral replication of influenza viruses [70, 71]. We short-listed 21 commercially available drugs targeting 16 GPCR and 5 ion channel genes required for A/WSN/33, A/CA/04/09, or B/Yamagata/16/1988 replication in A549 cells [27]. We used Ingenuity Pathway Analysis, Drugbank 3.0, the Drug Gene Interaction Database (DGIdg), and PubChem information to identify potential agonists or inhibitors drugs that directly affected the gene targets [27, 55–57]. Drug candidates were evaluated for their therapeutic potential to inhibit A/WSN/33 replication in A549 cells infected. The percent A/WSN/33 infected cells was determined at 12h and 24h post-treatment by Cellomics. Pretreatment with Clopidogrel or Triamterene reduced the percentage of influenza-infected cells at multiple time points (Fig 1). Inhibition was only significant at higher drug concentrations, but neither drug affected cell viability (S2 and S3 Figs). These findings prompted the further evaluation of Clopidogrel and Triamterene.

Clopidogrel is a small molecule thienopyridine antagonist which specifically and irreversibly binds to the purinergic receptor P2RY12 blocking adenosine 5’-diphosphate (ADP) binding although its exact mechanism is not fully understood [72]. Clopidogrel received FDA approval in late 1997 to reduce the risk of vascular and cerebrovascular disease [73]. We evaluated Clopidogrel pretreatment of Calu-3 cells for its ability to inhibit influenza replication. Calu-3 cells were pretreated with Clopidogrel for 24h or with LMB control for 2h before A/WSN/33 (MOI = 0.01) infection, and then the antiviral outcomes were determined by plaque assays, TCID_50_ assays, and the percentage of influenza-infected cells were determined. Based on these endpoint assays, Clopidogrel pretreatment consistently significantly, (p<0.05) reduced A/WSN/33 virus production using 50–125 uM concentrations (Fig 2A and 2B). Although pretreatment did not completely inhibit virus replication the results suggest that Clopidogrel treatment affected virus spread as shown by Cellomics image analysis (Fig 2C). Interestingly, NP staining of Clopidogrel treated cells showed that like LMB treatment NP staining was localized to DAPI-stained nuclei (Fig 2D). We further showed that host-directed antiviral therapy provides an antiviral strategy across strains and types of influenza viruses.

We show that Clopidogrel pretreatment is effective against circulating A/CA/04/09 influenza A virus and B/Yamagata/16/1988 strains (Fig 4). Clopidogrel pretreatment recapitulated previous results and significantly (p<0.05) inhibited A/CA/04/09 replication using 125–50 uM concentrations (Fig 4A and 4B). Importantly, the percent of A/CA/04/09 infected cells was significantly (p<0.05) reduced when treated using 50–125 uM concentrations (Fig 4C). Similarly, Clopidogrel pretreatment significantly (p<0.05) reduced B/Yamagata/16/1988 titers using 10–125 uM concentrations (Fig 4E and 4F) and reduced the percentage of infected cells at all concentrations and significantly (p<0.05) reduced with nearly all concentrations of drug (Fig 4G). These data show the antiviral potential of repurposing drugs targeting host genes.

The ability of Clopidogrel to inhibit A/WSN/33, A/CA/04/09, or B/Yamagata/16/1988 replication may be linked to inhibition of signaling pathways associated with P2RY12. Blocking P2RY12 with Clopidogrel improves outcome and protects the lung from severe injury following IAV infection in mouse models by reducing platelet activation [74]. We have previously shown that RNAi silencing of P2RY12 inhibits A/WSN/33, A/CA/04/09 and B/Yamagata/16/1988 replication demonstrating its importance during replication across influenza strains and types [27]. This GPCR purinergic receptor modulates the activation of the Raf/MEK/Erk pathway [46]. Inhibition of the Raf/MEK/Erk cascade during influenza replication leads to reduced influenza virus production and retention of vRNP within the nucleus [47, 48]. We confirmed that pretreatment with Clopidogrel localizes NP staining to the nucleus suggesting retention of NP-coated vRNPs in the nucleus as a mechanism inhibiting viral replication [75]. The observation that higher Clopidogrel concentrations are needed to achieve the NP staining phenotype and greater reduction in virus replication is likely because Clopidogrel is a pro-drug. It is thought that Clopidogrel undergoes sequential oxidative steps facilitated by cytochrome P450 enzymes to form an active metabolite [72, 76]. The requirement for activation is not clear as studies report that Clopidogrel is active *in vitro* without bioactivation [72, 77, 78]. Of note, it has been shown in animal studies that higher doses of Clopidogrel are needed for activity compared to the lower dosing requirements used in humans suggesting differences in metabolism [72]. It should be noted that *in vitro* data show a negative interaction between Clopidogrel and oseltamivir activation although these results have not been substantiated *in vivo* [79].

Triamterene is a diuretic that inhibits epithelial sodium channels (ENaC) [80] and was FDA-approved in 1964 for the treatment of edema [81]. The sodium channel gene SCNN1D was previously shown to be a pro-influenza host gene [27, 66, 82]. SCNN1D is not expressed in rodents limiting its evaluation [83]. We evaluated Triamterene pretreatment of Calu-3 cells for its ability to inhibit influenza replication. Calu-3 cells were pretreated with Triamterene for 24h, or with 10 nM LMB control for 2h before A/WSN/33 (MOI = 0.01) infection, then plaque assays, TCID_50_ assays, and the percentage of influenza-infected cells were determined. Based on these endpoint assays, pretreatment with Triamterene significantly (p<0.05) reduced A/WSN/33 replication using 100–125 uM concentrations (Fig 3A and 3B). The percent influenza infected Calu-3 cells was significantly (p<0.05) reduced following treatment using 0.5–125 uM concentrations (Fig 3C and 3D). We examined Triamterene pretreatment of Calu-3 cells on A/CA/04/09 and B/Yamagata/16/1988 replication (Fig 5). Triamterene pretreatment recapitulated previous results showing significant (p<0.05) inhibition of A/CA/04/09 replication using 100–125 uM concentrations (Fig 5A and 5B). The percent A/CA/04/09 infected cells was also significantly (p<0.05) reduced following pretreatment using 50–125 uM concentrations (Fig 5C). Triamterene pretreatment was effective in inhibiting B/Yamagata/16/1988 replication (Fig 5E), significantly (p<0.05) reduced HA titer using 10–125 uM concentrations (Fig 5F) and significantly (p<0.05) reduced the percentage of influenza-infected cells at all concentrations (Fig 5G). These results show the broad antiviral potential of repurposing Triamterene.

Triamterene targets epithelial sodium channels (EaNC), which are composed of 4 subunits (α, β, γ and δ), thus it is difficult to attribute the inhibitory effects to a specific subunit or action [84]. Based on our previous RNAi screen, it is likely that the δ subunit gene SCNN1D is contributing to limiting replication [27]. These results that are linked to Triamterene-induced changes in ion gradients and signaling cascades affect the membrane transport of proteins required for influenza replication. For example, influenza virus entry into the cell is regulated by a Ca^+2^ dependent signaling cycle which directly regulates clathrin-mediated or clathrin-independent endocytosis [85, 86]. As Clopidogrel and Triamterene are available by prescription, their use as repurposed drugs may be useful as an adjunct treatment for antiviral drug-resistant influenza strains.

## Supporting information

S1 Fig Clopidogrel and Triamterene do not reduce A549 viability. A CellTiter Blue assay was used to evaluate changes in A549 cell viability following 48h treatment with Clopidogrel or Triamterene. Results are shown as the mean percent of the DMSO-treated control ± standard error. Toxicity is defined as ≥20% loss of viability compared to mock control. Asterisks indicate significant differences from the DMSO treated control by one-way analysis of variance with Dunnett’s multiple-comparison test (P < 0.05).
(PDF)

S2 Fig Clopidogrel pretreatment does not reduce Calu-3 viability. A CellTiter Blue assay was used to evaluate Calu-3 cell viability. Following 48h treatment with Clopidogrel where the drug and media were replaced at 24h the mean percent of DMSO-treated control ± standard error was determined. Toxicity was defined as ≥20% loss of viability compared to the mock control. Asterisks indicate significant differences from the DMSO treated control by one-way analysis of variance with Dunnett’s multiple-comparison test (P < 0.05).
(PDF)

S3 Fig Triamterene does not reduce Calu-3 viability. CellTiter Blue non-destructive assay was used to evaluate changes in cell viability of CALU-3 cells following 48h treatment with Triamterene following a replenishment protocol where drug and media were replaced at 24h. Data is presented as mean of percentage of DMSO treated control ± standard error (Mean ± SEM). Toxicity is defined as >20% loss of viability compared to mock control. Asterisks indicate significant differences from the DMSO treated control by ordinary one-way analysis of variance with Dunnett’s multiple-comparison test (P < 0.05).
(PDF)

S1 File CellTiter Blue cell viability assay. (PDF)
