# Supplementary material for: Drug repositioning of Clopidogrel or Triamterene to inhibit influenza virus replication in vitro
Source: PLoS One. 2021 Oct 29;16(10):e0259129. doi: 10.1371/journal.pone.0259129 (PMC8555795; doi:10.1371/journal.pone.0259129)
Supplement: S1 Fig — A CellTiter Blue assay was used to evaluate changes in A549 cell viability following 48h treatment with Clopidogrel or Triamterene. Results are shown as the mean percent of the DMSO-treated control ± standard error. Toxicity is defined as ≥20% loss of viability compared to mock control. Asterisks indicate significant differences from the DMSO treated control by one-way analysis of variance with Dunnett’s multiple-comparison test (P < 0.05). (PDF) [file pone.0259129.s001.pdf]

Fig S1

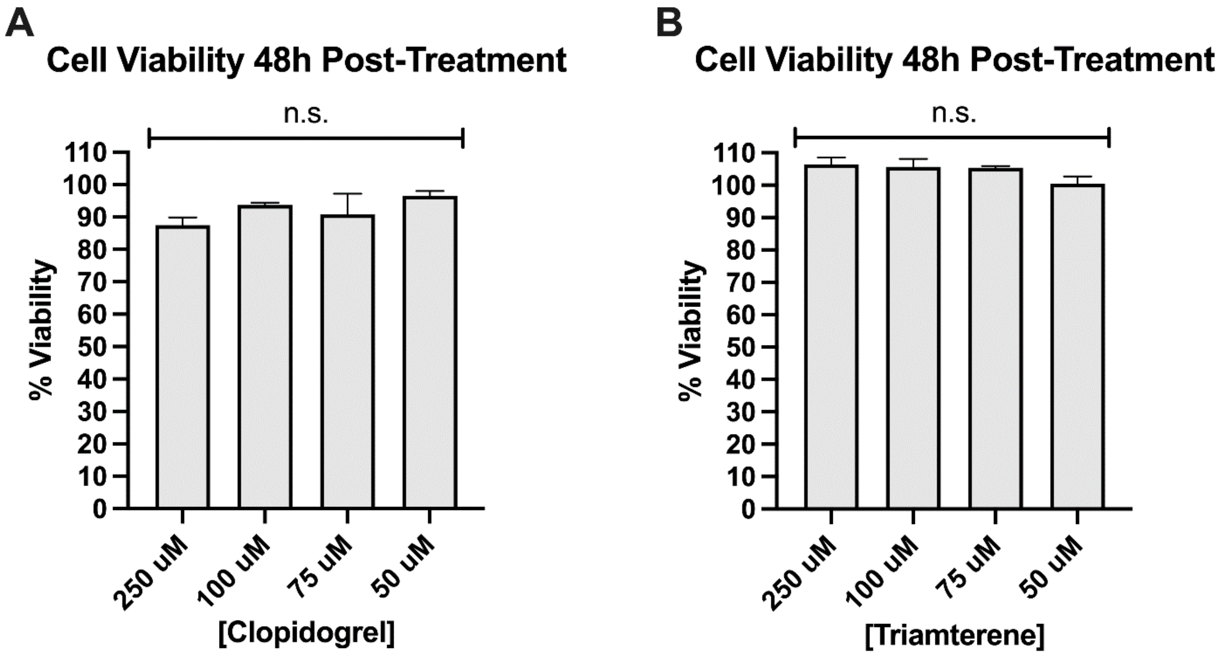

Fig S1. Clopidogrel and Triamterene do not reduce A549 viability*.* A CellTiter Blue assay was used to evaluate changes in A549 cell viability following 48h treatment with Clopidogrel or Triamterene. Results are shown as the mean percent of the DMSO-treated control ± standard error. Toxicity is defined as $\geq$20% loss of viability compared to mock control. Asterisks indicate significant differences from the DMSO treated control by one-way analysis of variance with Dunnett's multiple-comparison test ($P < 0.05$).
