# Supplementary material for: Drug repositioning of Clopidogrel or Triamterene to inhibit influenza virus replication in vitro
Source: PLoS One. 2021 Oct 29;16(10):e0259129. doi: 10.1371/journal.pone.0259129 (PMC8555795; doi:10.1371/journal.pone.0259129)
Supplement: S2 Fig — A CellTiter Blue assay was used to evaluate Calu-3 cell viability. Following 48h treatment with Clopidogrel where the drug and media were replaced at 24h the mean percent of DMSO-treated control ± standard error was determined. Toxicity was defined as ≥20% loss of viability compared to the mock control. Asterisks indicate significant differences from the DMSO treated control by one-way analysis of variance with Dunnett’s multiple-comparison test (P < 0.05). (PDF) [file pone.0259129.s002.pdf]

Fig S2

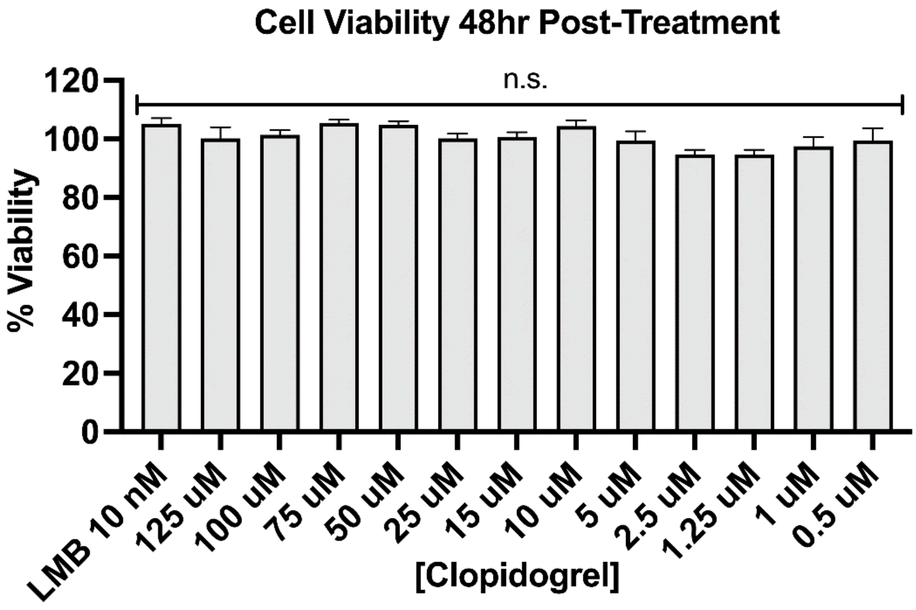

Fig S2. Clopidogrel pretreatment does not reduce Calu-3 viability. A CellTiter Blue assay was used to evaluate Calu-3 cell viability. Following 48h treatment with Clopidogrel where the drug and media were replaced at 24h the mean percent of DMSO-treated control ± standard error was determined. Toxicity was defined as $\geq$20% loss of viability compared to the mock control. Asterisks indicate significant differences from the DMSO treated control by one-way analysis of variance with Dunnett’s multiple-comparison test ($P < 0.05$).
