# Supplementary material for: Drug repositioning of Clopidogrel or Triamterene to inhibit influenza virus replication in vitro
Source: PLoS One. 2021 Oct 29;16(10):e0259129. doi: 10.1371/journal.pone.0259129 (PMC8555795; doi:10.1371/journal.pone.0259129)
Supplement: S3 Fig — CellTiter Blue non-destructive assay was used to evaluate changes in cell viability of CALU-3 cells following 48h treatment with Triamterene following a replenishment protocol where drug and media were replaced at 24h. Data is presented as mean of percentage of DMSO treated control ± standard error (Mean ± SEM). Toxicity is defined as >20% loss of viability compared to mock control. Asterisks indicate significant differences from the DMSO treated control by ordinary one-way analysis of variance with Dunnett’s multiple-comparison test (P < 0.05). (PDF) [file pone.0259129.s003.pdf]

Fig S3

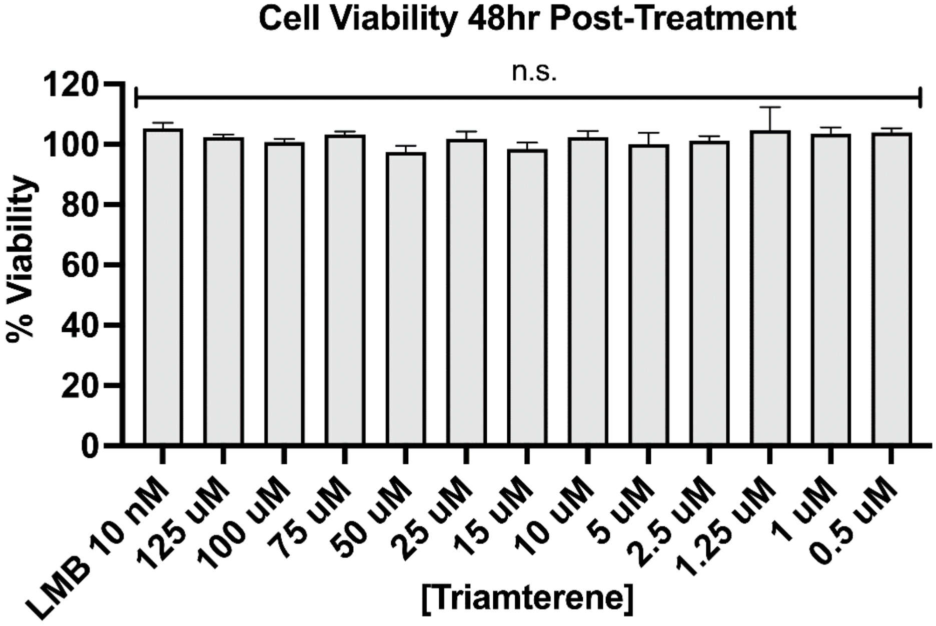

Fig S3. Triamterene does not reduce Calu-3 viability. CellTiter Blue non-destructive assay was used to evaluate changes in cell viability of CALU-3 cells following 48h treatment with Triamterene following a replenishment protocol where drug and media were replaced at 24h. Data is presented as mean of percentage of DMSO treated control ± standard error (Mean ± SEM). Toxicity is defined as >20% loss of viability compared to mock control. Asterisks indicate significant differences from the DMSO treated control by ordinary one-way analysis of variance with Dunnett's multiple-comparison test ($P < 0.05$).
