# Supplementary material for: Drug repositioning of Clopidogrel or Triamterene to inhibit influenza virus replication in vitro
Source: PLoS One. 2021 Oct 29;16(10):e0259129. doi: 10.1371/journal.pone.0259129 (PMC8555795; doi:10.1371/journal.pone.0259129)
Supplement: S1 File — (PDF) [file pone.0259129.s004.pdf]

Supporting Methods

CellTiter Blue cell viability assay

The selected drugs were evaluated for cytotoxicity using A549 cells and Calu-3 cells using a CellTiter blue cell viability assay (Promega, WI). Drugs which did not cause cytotoxicity (<20%) when compared to the DMSO control were further evaluated. Briefly, 1.5 x 104 A549 or Calu-3 cells were seeded into 96-well flat-bottom plates (Costar) and incubated for the relevant timepoints at 37oC/5% CO2. Subsequently, the cells were gently washed 1x with PBS (GIBCO), and minimum essential media (MEM; HyClone, Logan, UT) supplemented with 0.3% (v/v) bovine serum albumin (BSA; Gibco, Waltham, Massachusetts) was added to the plates and plates placed at 37oC/5% CO2. Drug stocks were prepared in filter-sterilized DMSO (Sigma) to a stock concentration of 10 mM. Drugs were dispensed into 96-well plates using a D300 BioPrinter digital drug dispenser (HP, Palo Alto, CA) in MEM supplemented with 0.3% BSA to final concentrations of 500, 200, 150 and 100 for A549 cells and 250, 200, 150, 100, 50, 30, 20, 10, 5, 2.5, 2 and 1 uM for Calu3 cells. Dilutions were transferred to A549 or Calu-3 plates for final drug concentrations of 250, 100, 75, and 50 uM and 125, 100, 75, 50, 25, 15, 10, 5, 2.5, 1.25, 1, and 0.5 uM, respectively. All wells were normalized to 1% DMSO for A549 cell evaluation, and 0.833% DMSO for Calu-3 cell evaluation. Cells were incubated for the relevant timepoints at 37°C/5% CO2. Following incubation, CellTiter blue reagent was added to each well and incubated at 37°C/5% CO2 for 2 h. The absorbance of the plates was determined using a spectrophotometer plate reader (Tecan Trading; AG, Switzerland) at 570 nm with reference at 600 nm. Percent viability was determined by comparing the DMSO control to drug- treated cells.
